# Editorial: ML and AI Safety, Effectiveness and Explainability in Healthcare

**DOI:** 10.3389/fdata.2021.727856

**Published:** 2021-07-12

**Authors:** David Benrimoh, Sonia Israel, Robert Fratila, Caitrin Armstrong, Kelly Perlman, Ariel Rosenfeld, Adam Kapelner

**Affiliations:** ^1^ Department of Psychiatry, McGill University, Montreal, QC, Canada; ^2^ Aifred Health, Inc., Montreal, QC, Canada; ^3^ Department of Information Science, Bar-Ilan University, Ramat Gan, Israel; ^4^ Department of Mathematics, Queens College (CUNY), New York City, NY, United States

**Keywords:** healthcare, artificial inteligence, machine learning, safety, effectiveness, explainability

The increasing performance of machine learning and artificial intelligence (ML/AI) models has led to them being encountered more frequently in daily life, including in clinical medicine (Bruckert et al.; Rosenfeld et al., 2021). While concerns about the opaque “black box” nature of ML/AI tools are not new, the need for practical solutions to the interpretability problem has become more pressing as ML/AI devices move from the laboratory, through regulatory processes that have yet to fully catch up to the state-of-the-art (Benrimoh et al., 2018a), and to the bedside. This special edition targets three key domains in which innovation and clearer best practices are required for the implementation of ML/AI approaches in healthcare: ensuring safety, demonstrating effectiveness, and providing explainability. Notably, the first two have long been staples in the evaluation of drugs and medical devices (i.e., in order to be approved for human use, products must prove that they are safe and effective—often compared to a reasonable comparator) (Spławiński and Kuźniar, 2004). The third requirement—that of explainability—appears to be unique to ML/AI, due to the challenge of explaining how models arrive at their increasingly accurate conclusions. Yet, upon closer examination, one might argue that the explainability criterion has been implied in the past: mechanisms of action of drugs and devices are generally described in their product documentation (Health Canada, 2014). However, this can be misleading. For instance, many drugs have known receptor binding profiles and putative mechanisms of actions, although the precise mechanisms by which they produce their effect remain unclear despite their widespread use in clinical practice. Prime examples of this are lithium (Shaldubina et al., 2001) and electroconvulsive therapy (Scott, 2011), both longstanding and highly effective treatments whose mechanisms of action remain controversial. Indeed, even the precise mechanism of general anesthesia is a subject of debate (Pleuvry, 2008). As such, we must consider a compromise-that of *sufficient* explainability (Clarke and Kapelner). This involves answering the question: how much must we know about a model in order to determine that it is safe to use in clinical practice? The articles in this special edition begin to explore possible answers to this as well as other key questions in the application of ML/AI to healthcare contexts.

Bruckert et al. propose a Comprehensible Artificial Intelligence (cAI) framework, which they describe as a “cookbook” approach for integrating explainability into ML/AI systems intended to support medical decision-making. Notably, the authors do not limit explainability to an understanding of general rules a model might use to make predictions, but rather extend it to an example-level approach where human-interpretable semantic information is passed from the machine to the human user. They also discuss systems which not only provide an explanation to the user, but which receive feedback on this explanation in order to learn the implicit rules which experts may use as part of their routine decision-making. Future research could examine the potential biases which a machine might learn from experts, and how this could be mitigated.

Clarke and Kapelner present a Bayesian ML/AI model that predicts outcomes after lens implant (a surgical treatment for cataracts). Their approach to explainability entails generating a list of the most important features used by the model. This method coheres with pre-ML/AI approaches in ophthalmology, which relied on traditional linear equations with clear variables (e.g. Dang and Raj, 1989), an example of an explainability already standard in the field. Because their approach is Bayesian, their predictions come with uncertainty intervals. Thus, the more uncertain the prediction, the wider the interval. Similar to Bruckert et al., the authors insist upon a “human in the loop,” noting that surgeons ought to use the algorithm’s predictions and uncertainty intervals as a guide within the context of their clinical judgement. Further, they note that the consequences of an incorrect prediction are relatively minor and simply require corrective medical procedures already employed in standard practice. It is interesting to consider that these kinds of predictions - in which a correct prediction provides a benefit but where a failed prediction carries low risk - are ideal first applications of ML/AI while they remain novel technologies (Benrimoh et al., 2018b).

Desai et al. introduce a ML/AI model for the identification of suicidal ideation in the general population. Using a sensitivity analysis, they demonstrate that a deep learning model, which is traditionally difficult to interpret, can be queried using standard statistical approaches to determine whether relationships between variables and outcomes identified by the model are coherent with the literature. Doing so not only makes deep learning a more viable model architecture for use in healthcare, but in situations where a large body of literature exists allows for a “sanity check” of each model produced to ensure they have not learned “quirks” of a biased or non-representative dataset. This could help address Bruckert et al.’s concern about poor explainability arising from biased data and models.

Finally, Wong et al. offer a comprehensive discussion of challenges and opportunities for machine learning approaches in acute respiratory failure. Their discussion is a prime example of the level of granularity necessary for content experts to provide when building AI/ML models in healthcare. In addition to a general review of ML/AI concepts, the authors discuss domain-specific sources of bias as well as difficulties in operationalizing the outcomes that models should be trained to predict. This discourse serves as a useful entry point to the special issue for the clinically-oriented reader who is less familiar with ML/AI approaches. This article reminds us that each healthcare domain has unique challenges, meaning that a one-size-fits-all approach to explainability and the evaluation of safety and effectiveness is unlikely to succeed.

This special edition provides the reader with both a survey of current approaches to integrating ML/AI into healthcare as well as in-depth discussions of how to determine model safety, measure model effectiveness, and provide model explainability that will be of use to clinicians and regulators.
